# Digestibility, rumen fermentation, and blood metabolites of Barki rams fed *Panicum maximum* cv. Mombasa hay as a substitute for alfalfa hay, with or without spirulina

**DOI:** 10.1007/s11250-026-04926-w

**Published:** 2026-03-17

**Authors:** M. I. Meteab, M. M. Khorshed, M. S. Nassar, Abeer. M. EL-Essawy, N. E. El-Bordeny

**Affiliations:** 1 Animal and Poultry Nutrition Dept., Animal and Poultry Divi, Desert Research Center, Mataryia, Cairo Egypt; 2 Animal Production Dept, Fac. of Agric, Ain-Shams Univ, 68 Hadayek Shoubra, Cairo, 111241 Egypt

**Keywords:** *Panicum maximum*, Alfalfa, Spirulina, Barki rams, Digestibility trials, Rumen fermentation

## Abstract

There is a growing interest in alternative feed sources for sheep, especially under desert conditions. This study evaluated the effects of replacing 25% of alfalfa hay with *Panicum maximum* hay in three isocaloric and isonitrogenous diets, with or without Spirulina supplementation (2 mg/g), on nutrient digestibility and rumen fermentation in Barki rams. Forty-eight adult rams (2 years old; 45 ± 0.60 kg) were randomly assigned to six groups (*n* = 8). Forage represented 40% of the diet, and its composition varied as follows: 0% Panicum (all alfalfa with no replacement; T1, T2), 25% of the forage as Panicum (partial replacement; T3, T4), and 100% of the forage as Panicum (complete replacement; T5, T6). Spirulina was added to T2, T4, and T6. Results showed that all alfalfa hay diets significantly (*P* < 0.0001) enhanced rumen total volatile fatty acids (TVFA), ammonia (NH_3_) concentration, nutrient digestibility, nitrogen balance, and overall nutrient composition compared to diets with 10% Panicum and 30% alfalfa or 40% Panicum. Moreover, Spirulina supplementation significantly (*P* < 0.0001) improved all measured rumen parameters and digestibility indices relative to non-supplemented groups. Regarding blood metabolites, rams fed by 40% alfalfa hay exhibited the highest (*P* < 0.0001) levels of total protein, albumin, globulin, and urea, followed by those receiving 10% Panicum and 30% alfalfa. Spirulina supplementation also significantly increased these blood parameters compared with the unsupplemented groups. In conclusion, partial replacement of alfalfa hay with Panicum maximum hay (25%), combined with Spirulina supplementation, can enhance rumen fermentation, nutrient utilization, and metabolic profiles in Barki rams, suggesting a promising strategy for improving productivity under forage-limited conditions.

## Introduction

Egypt’s agricultural sector faces increasing pressure, including rapid population growth and climate change, which have resulted in severe water scarcity and reduced availability of conventional feed resources (Amer et al., 2017). Agriculture accounts for approximately 85% of total water consumption in Egypt, making the identification of alternative forage resources with improved water-use efficiency a critical priority for sustainable livestock production (Amer et al., 2017). Consequently, there is a growing interest in enhancing ruminant productivity under arid and semi-arid conditions, which is negatively impacted by the adverse effects of global climate change on water and feed availability (Poppi and McLennan 2010).

Alfalfa is widely regarded as one of the best forage crops, often referred to as the “queen of forages.” due to its high protein content, essential minerals, vitamins, and low cellulose content, which contributes to excellent digestibility, and enhance rumen fermentation and improve animal productivity (Rezaeian et al. 2020; Zhang and Wang 2025; Wang et al. 2025). However, alfalfa production is associated with very high water requirements, ranging from 21,420 to 24,000 m³/ha/year under Egyptian conditions (Amer et al. 2017).

In contrast, *Panicum maximum cv. Mombasa* has been identified as a promising forage alternative for arid regions due to its high biomass yield, palatability, rapid growth rate, and tolerance to drought and salinity (Adjolohoun et al. 2013; Hare et al. 2014). Under Egyptian desert conditions, *Panicum maximum* requires approximately 8,330 m³/ha/year, which is substantially lower than alfalfa (El Wardany et al. 2022). Although its annual dry matter yield (approximately 33 t/ha) is lower than that of alfalfa (approximately 81.7 t/ha), *Panicum maximum* demonstrates acceptable nutritional quality, particularly under moderate water stress and nitrogen fertilization (Freitas et al. 2007; Hosni et al. 2022; Alsunaydi et al. 2024).

Several studies conducted under conditions similar to those of Egypt have reported that partial replacement of forage legumes like alfalfa hay with *Panicum maximum* hay can maintain or improve forage quality and nutrient digestibility (Alasa et al. 2014). However, the nutritive value of Panicum maximum is strongly influenced by plant maturity, with advancing maturity leading to reductions in crude protein and ether extract and increases in neutral detergent fiber (NDF) and acid detergent fiber (ADF), due to lignification and structural carbohydrate accumulation (Gándara et al. 2017). These limitations highlight the need for nutritional strategies that improve the utilization efficiency of Panicum-based diets.

In this context, microalgae supplementation, particularly Spirulina (Arthrospira platensis), has gained attention as a functional feed additive in ruminant nutrition (Molino et al. 2018). Spirulina is characterized by its high crude protein content (65–70% of dry matter), balanced amino acid profile, vitamins, minerals, carotenoids, and bioactive compounds such as γ-linolenic acid and phenolic compounds (Farag et al. 2016; Mariey et al. 2012). These components have been shown to stimulate ruminal microflora activity, modulate gut microbiota composition, and enhance fermentation efficiency (Gotteland et al. 2020; Rabee et al. 2022). Previous studies have reported increases in ruminal total volatile fatty acids (TVFA) and ammonia concentrations following Spirulina supplementation in ruminant diets (Panjaitan et al. 2014; Costa et al. 2016; Gaafar et al. 2017).

Recent evidence, including a meta-analysis by Firdaus et al. (2025), indicates that Spirulina supplementation improves growth performance, rumen fermentation, antioxidant capacity, and metabolic health in small ruminants without adverse effects on blood biochemical parameters. In our previous in vitro study (Meteab et al. 2025), replacing 25% of alfalfa hay with Panicum maximum hay, combined with Spirulina supplementation at 2 mg/g of feed, resulted in the highest nutrient degradability and gas production compared with higher substitution levels.

Based on these findings, the present study aimed to evaluate the effects of partial (25%) and complete (100%) replacement of alfalfa hay with Panicum maximum hay, with or without Spirulina supplementation (2 mg/g), on rumen fermentation characteristics, nutrient digestibility, nitrogen balance, and blood biochemical parameters in Barki rams under Egyptian conditions.

## Materials and methods

This research was conducted in Maryout Research Station (N 30°09’07.5024^”^, E 31^°^14’28.9824”), and Animal Nutrition Department labs, Desert Research Center (N 30°07’18.0372”, E 31°18’54.3276”), both are affiliated with the Desert Research Center (DRC), Cairo, Egypt.

### Feed materials and additives

Alfalfa (*Medicago sativa*) was harvested from Maryout Station. *Panicum maximum cv. (Mombasa*) was harvested from Al-Maghrah (N 30°15’10.9944^”^, E 28^°^55’55.0056”), Matrouh Governorate. The collected plants from Alfalfa and *Panicum maximum* hay were sun-dried (at 28 °C and 33% humidity) for 6 days until the hays were obtained.

Alfalfa and *Panicum maximum* hay were chopped and stored in the form of bales until used in the metabolic experiment. Spirulina extract (100% pure) in powdered form was purchased from the Algae Biotechnology Unit (National Research Centre, Egypt).

### Animals, feeding, and experimental design

Forty-eight clinically healthy adult Barki Rams were used in a metabolic experiment. The animals were 2 years of age, with an initial live body weight of 45 ± 0.60 kg, and were selected from the Maryout Research Station flock. Prior to the experiment, all rams were subjected to routine veterinary examination and housed under uniform management and environmental conditions. The experiment was designed as a 3 × 2 factorial arrangement to evaluate the effect of forage source and spirulina supplementation. The factors consisted of three levels of alfalfa hay replacement with panicum hay (0%, 25%, and 100%) in isocaloric and isonitrogenous diets, and two levels of spirulina (0 and 2 mg/g of feed). All diets contained 40% forage, in which Panicum hay was used to partially or fully substitute alfalfa hay, and 60% concentrate mixture. To maintain comparable dietary energy and protein contents across treatments, the concentrate feed mixtures were adjusted accordingly, resulting in three concentrate formulations that differed slightly in composition but supported equivalent nutrient supply.

### This design produced six treatment combinations as follows

T1 (Control): 40% alfalfa hay (0% *Panicum maximum*), 60% concentrate feed mixture 1 (Diet 1), without Supplementation.

T2: 40% alfalfa hay (0% *Panicum maximum*), 60% concentrate feed mixture 1 (Diet 1), supplemented with spirulina at 2 mg/g diet.

T3: 25% of forage replacement (30% alfalfa hay and 10% *Panicum maximum* hay), 60% concentrate feed mixture 2 (Diet 2), without Supplementation.

T4: 25% of forage replacement (30% alfalfa hay and 10% *Panicum maximum* hay), 60% concentrate feed mixture 2 (Diet 2), supplemented with spirulina at 2 mg/g diet.

T5: 40% *Panicum maximum* hay (100% forage replacement), 60% concentrate mixture 3 (Diet 3), without supplementation.

T6: 40% *Panicum maximum* hay(100% forage replacement), 60% concentrate mixture 3 (Diet 3), supplemented with spirulina at 2 mg/g diet.

The rams were weighed and randomly allocated into six equal groups (*n* = 8 per group), with each group assigned to one of the experimental diets. All rams were housed under uniform management and environmental conditions throughout the experimental period. The three selected levels of alfalfa hay replacement with panicum hay (0%, 25%, and 100%) and the two levels of spirulina (0 and 2 mg/g of feed) were based on our preliminary pilot in vitro study (Meteab et al. 2025). The experimental design, diet formulation, and chemical compositions are detailed in Table 1.

**Table 1:** Experimental design, ingredients of the basal diet/100 kg DM, and chemical composition of the experimental dietary formulations used in the metabolic trial

		T1	T3	T5	T2	T4	T6	Alfalfa	Panicum	Spirulina
Items		Experimental design								
Factor (1): 40% Forage	Alfalfa hay	100%	75%	0%	100%	75%	0%	**-**	**-**	**-**
	Panicum hay	0%	25%	100%	0%	25%	100%	**-**	**-**	**-**
Factor (2): Spirulina (mg/g)		0	0	0	2	2	2	**-**	**-**	**-**
60% concentrate feed mixture (CFM)		CFM1	CFM2	CFM3	CFM1	CFM2	CFM3	**-**	**-**	**-**
Number of rams		8	8	8	8	8	8	**-**	**-**	**-**
Duration of the experiment (days)		28	28	28	28	28	28	**-**	**-**	**-**
**Ingredients**		**Ingredients of concentrate feed mixture (CFM)**							**-**	**-**
Alfalfa hay (A), kg/100kg		40.00	30.00	0.00	40.00	30.00	0.00	**-**	**-**	**-**
*Panicum maximum* (P), kg/100kg		0.00	10.00	40.00	0.00	10.00	40.00	**-**	**-**	**-**
Corn, kg/100kg		32.80	30.90	27.90	32.80	30.90	27.90	**-**	**-**	**-**
Soya meal, kg/100kg		9.50	15.30	21.60	9.50	15.30	21.60	**-**	**-**	**-**
Wheat bran, kg/100kg		15.60	11.70	8.40	15.60	11.70	8.40	**-**	**-**	**-**
Salt, kg/100kg		0.90	0.90	0.90	0.90	0.90	0.90	**-**	**-**	**-**
Limestone, kg/100kg		0.90	0.90	0.90	0.90	0.90	0.90	**-**	**-**	**-**
Premix, kg/100kg		0.30	0.30	0.30	0.30	0.30	0.30	-	-	-
**Chemical composition of the experimental dietary formulations used in the metabolic trial (g/kg DM basis)**										
Ash, g/Kg		89	89	91	91	99	99	113	139	131
Dry matter, g/Kg		887	887	887	887	877	877	884	895	914
Organic Matter, g/Kg		911	911	909	909	901	901	887	861	869
Crude Fiber, g/Kg		160	159	162	162	167	167	333	353	60
Crude Protein, g/Kg		168	168	167	168	167	167	169	80	472
Ether Extract, g/Kg		23	23	24	24	26	26	5	11	38
Nitrogen-Free Extract, g/Kg		560	561	556	555	541	541	380	417	299
Neutral Detergent Fiber, g/Kg		354	353	355	355	382	382	474	546	398
Acid Detergent Fiber, g/Kg		154	154	155	155	171	171	311	352	189
Non-Fiber Carbohydrate, g/Kg		367	367	364	363	326	326	239	270	-
**Calculated energy contents**										
Gross Energy, kcal/ kg DM		3766	3765	3736	3766	3764	3737	3574.25	3685.75	3675.50
Digestible Energy, kcal/ kg DM		2480	2480	2460	2480	2480	2460	2351.48	2424.84	2418.09
Metabolizable Energy, kcal/ kg DM		2030	2030	2020	2030	2030	2020	1928.21	1988.37	1982.84

T1 (Control): 0% *Panicum maximum* (40% alfalfa hay) + no Supplementation, T2: 0% *Panicum maximum* (40% alfalfa hay) + 2 mg/g Spirulina, T3: 25% *Panicum maximum* (30% alfalfa hay + 10% *Panicum maximum* hay) + no Supplementation, T4: 25% *Panicum maximum* (30% alfalfa hay + 10% *Panicum maximum* hay) + 2 mg/g Spirulina, T5: 100% *Panicum maximum* (40% *Panicum maximum* hay) + no Supplementation, T6: 100% *Panicum maximum* (40% *Panicum maximum* hay) + 2 mg/g Spirulina. DE, ME, and GE are calculated according to NRC (2001)

### Metabolic trial and sample collection procedures

The experiment was conducted in two sequential phases: an adaptation phase followed by a sampling (metabolic) phase. During the adaptation phase (21 days), all rams were housed in individual pens under uniform management conditions to allow physiological adaptation to the experimental diets. Animals were fed twice daily at 07:00 and 14:00 h, with free access to drinking water. Feed allowances were provided according to Kearl (1982) maintenance requirements, and routine veterinary care was maintained throughout the experimental period. Following the adaptation period, rams were transferred to individual steel metabolic crates for the sampling phase (7 days). The crates (approximately 1.5 m × 0.7 m) were designed to enable separate and quantitative collection of feces and urine. The first 2 days in the crates were used for acclimatization, followed by 5 consecutive days of total feces and urine collection. During the collection period, total fecal output was recorded daily, thoroughly mixed, and a representative 10% subsample was retained for subsequent chemical analysis. Total urine volume was measured daily, and 10% of the daily urine output was preserved in airtight containers containing 10% sulfuric acid to prevent nitrogen losses as ammonia. Urine samples were stored at 4 °C until nitrogen determination. Body weight of the rams was recorded after a 12-hour fasting period prior to morning feeding at the beginning (day 0) and end (day 28) of the experimental period.

### Chemical analysis

Representative samples of feeds, feed orts, and feces for each animal were oven-dried at 65 °C for 72 h, then ground in a cutter-type mill with a 1 mm screen. The samples were then subjected to a proximate chemical analysis, including total nitrogen, crude fiber (CF), ether extract (EE), and ash according to AOAC (2016). Additionally, crude protein (CP) was calculated, and nitrogen-free extract (NFE) was calculated by difference. Total nitrogen in urine was determined using the micro-Kjeldahl method according to AOAC (2016). Furthermore, neutral detergent fiber (NDF), acid detergent fiber (ADF), and acid detergent lignin (ADL) were determined according to sequential procedures outlined by Van Soest et al. (1991), using the Ankom200 (Ankom Technology Corp., Fairport, NY) filter bag technique.

### Rumen liquor samples

At the end of the collection period, samples of rumen liquor were taken at zero time (before feeding), 3, and 6 h post-feeding using a stomach tube, then filtered through four layers of cheesecloth. The pH value was determined directly in rumen liquor using the digital pH meter model Orian 680. Total VFA was determined by the steam distillation method using the Markham micro distillation unit (Warner, 1964). Ammonia (NH_3_) was determined by Nessler’s method as described by Meteab et al. (2025).

### Blood serum samples

Samples of blood were taken pre-feeding at the end of the 7-day collection period of the metabolic trial. Blood samples were drawn from the jugular vein of rams using a sterile needle into clean and dry serum tubes and centrifuged at 4000 rpm for 15 min. All serum samples were analyzed for total protein (TP) and Albumin (Alb) according to Reinhold (1953). Globulin (Glo) was calculated by subtracting serum albumin from serum total protein. Urea was determined according to Fawcett and Soctt (1960), Creatinine was determined according to Bartels et al. (1972), Cholesterol was determined according to Richmond (1973), Triglycerides were determined according to Fossati and Prencipe (1982). Alanine aminotransferase (ALT) and Aspartate aminotransferase (AST) were determined according to Reitman and Frankel (1957). All kits used were purchased from Human Co. (Germany) using the spectrophotometers Jenway 6100.

### Phytochemical screening

Qualitative phytochemical screening and quantitative estimation of total tannins, saponins, and total phenols as the major Anti-Nutritional Factors (ANF) were performed on all feed ingredients using the alcoholic extracts of alfalfa, *Panicum maximum*, and Spirulina, following the methods of Makkar et al. (1993) and Tava and Avato (2006). The results are presented in Table 2.

**Table 2:** Preliminary phytochemical screening and quantitative Estimation of the anti-nutritional factors (ANF) in the experimental ingredients

Type	Total tannins mg	Saponins g/100gDM	Total phenol %
Alfalfa hay	++	++	++
*Panicum maximum* hay	+++	ND	+++
*Spirulina*	+	+++	+
**Concentrations of the anti-nutritional factors (ANF) in the experimental ingredients**			
Alfalfa hay	2.58	2.64	0.20
*Panicum maximum* hay	3.75	ND	0.25
*Spirulina*	1.09	3.66	0.17

+: present, ND: Not Detected

### Statistical analysis

The experiment was designed as a factorial experiment (3 treatment diets × 2 spirulina supplementation levels) in a completely randomized design. Data were statistically analyzed using the SAS software (SAS, 2011). Significance was determined at the 5% level for all analyses. Mean Separation was conducted according to Duncan’s multiple-range test (Duncan 1955). Digestibility, nitrogen balance, and blood parameter data were analyzed using a two-way analysis of variance with interaction, according to the following General Linear Model.

$$\:{\mathrm{Y}}_{\mathrm{i}\mathrm{j}}={\upmu\:}\hspace{0.17em}+{\mathrm{T}}_{\mathrm{i}}\:+{\mathrm{S}}_{\mathrm{j}}+{+\left(\mathrm{S}\mathrm{*}\mathrm{T}\right)}_{\mathrm{i}\mathrm{j}}+{\mathrm{e}}_{\mathrm{i}\mathrm{j}}$$

Where Y_ij_ is the observation for the I^th^ treatment, µ: o```verall mean, T_i_: effect of the I^th^ treatment diet, S_j_: effect of the supplementation, (S*T) ij: the effect of the interaction between factors I and J, and eij: the random experimental error (0, σ^2^e).

The data on rumen fermentation parameters were analyzed using the MIXED procedure of SAS according to the following mixed Model.

$$\begin{aligned}\:{\mathrm{Y}}_{\mathrm{i}\mathrm{j}\mathrm{k}\mathrm{l}}&={\upmu\:}+{\mathrm{A}}_{\mathrm{i}}+{\mathrm{B}}_{\mathrm{j}}{+(\mathrm{A}\times\:\mathrm{B})}_{\mathrm{i}\mathrm{j}}+{\mathrm{T}}_{\mathrm{k}}+{(\mathrm{A}\times\:\mathrm{T})}_{\mathrm{i}\mathrm{k}}\cr&+{(\mathrm{B}\times\:\mathrm{T})}_{\mathrm{j}\mathrm{k}}+{(\mathrm{A}\times\:\mathrm{B}\times\:\mathrm{T})}_{\mathrm{i}\mathrm{j}\mathrm{k}}+{\mathrm{A}\mathrm{n}\mathrm{i}\mathrm{m}\mathrm{a}\mathrm{l}}_{\left(\mathrm{i}\mathrm{j}\right)}+{\varepsilon}_{\mathrm{i}\mathrm{j}\mathrm{k}\mathrm{l}}\end{aligned}$$

Where Y_ijkl_ represents the K^th^ Observation on the diet subjected to factors A and B, µ: overall mean, A_i_: fixed effect of substitution level (i = 0, 25, and 100%), B_j_: fixed effect of supplementation (j = 0 and 2 mg/), (A×B)_ij_: interaction between factors A and B, T_k_: fixed effect of time (k = repeated measurement occasions), Animal_(ij)_: random effect of animal nested within the A×B combination (subject term for repeated measures) and, ε_ijkl_: random experimental error.

## Results

The data of chemical composition (Table 1) clearly showed that there is a difference in the chemical composition between the Alfalfa hay and *Panicum maximum* hay, especially the crude protein (CP), as the CP in Alfalfa hay was 169 g/kg compared to 80 g/kg in the *Panicum maximum* hay. These differences in chemical composition, particularly the protein percentage, necessitated the formulation of experimental diets to be isonitrogenous and isocaloric.

### Rumen fermentation parameters

The impact of substituting alfalfa hay with *Panicum maximum* hay, with or without Spirulina supplementation (2 mg/g), on rumen fermentation parameters, total volatile fatty acids (TVFA), ammonia (NH₃), and pH was presented in Table 3. All measured values remained within the physiological range for healthy ruminants.

**Table 3:** The effect of substituting alfalfa hay with *Panicum maximum* hay with or without spirulina addition on rumen fermentation parameters during metabolic trial

Forage contents	TRT	Spirulina, (S) mg/g	Time	TVFA, mequ/dl	Ammonia, mg/ dl	pH value
100% A	T1	0.0 mg/g	0 Time	5.53^h^	15.01^feg^	6.85
			3 PF	9.83^ab^	19.8^b^	6.37
			6 PF	7.9^de^	17.35^d^	6.5
	T2	2.0 mg/g	0 Time	6.03^g^	18.92^cb^	6.65
			3 PF	10.17^a^	21.21^a^	6.15
			6 PF	9.47^b^	19.17^cb^	6.34
75% A + 25% P	T3	0.0 mg/g	0 Time	5.1^ih^	14.14^gh^	6.94
			3 PF	7.77^de^	17.53^d^	6.39
			6 PF	6.53^f^	15.42^fe^	6.56
	T4	2.0 mg/g	0 Time	5.3^h^	15.11^fe^	6.75
			3 PF	8.43^c^	18.8^c^	6.27
			6 PF	8.1^cd^	17.27^d^	6.41
100% P	T5	0.0 mg/g	0Time	4.7^i^	13.04^i^	6.82
			3 PF	6.73^f^	15.19^fe^	6.22
			6 PF	6.33^gf^	13.64^ih^	6.48
	T6	2.0 mg/g	0 Time	5.13^ih^	14.7^fg^	6.54
			3 PF	7.77^ed^	17.79^d^	6.13
			6 PF	7.5^e^	15.78^e^	6.33
Standard error				0.0916	0.1680	0.0256
P value		Substituting (A)		< 0.0001	< 0.0001	0.0067
		Spirulina (B)		< 0.0001	< 0.0001	< 0.0001
		A * B		0.9480	0.0374	0.8432
		Time		<0.0001	< 0.0001	< 0.0001
		A*time		<0.0001	0.0897	0.4169
		B*Time		< 0.0001	0.4477	0.1546
		A * B*Time		0.0165	0.0010	0.3972

A: Alfalfa Hay - P: Panicum Hay - S: Spirulina. T1 (Control): 0% *Panicum maximum* (40% alfalfa hay) + no Supplementation, T2: 0% *Panicum maximum* (40% alfalfa hay) + 2 mg/g Spirulina, T3: 25% *Panicum maximum* (30% alfalfa hay + 10% *Panicum maximum* hay) + no Supplementation, T4: 25% *Panicum maximum* (30% alfalfa hay + 10% *Panicum maximum* hay) + 2 mg/g Spirulina, T5: 100% *Panicum maximum* (40% *Panicum maximum* hay) + no Supplementation, T6: 100% *Panicum maximum* (40% *Panicum maximum* hay) + 2 mg/g Spirulina. PF: Post-feeding. TRT: Treatments

**Fig. 1:**
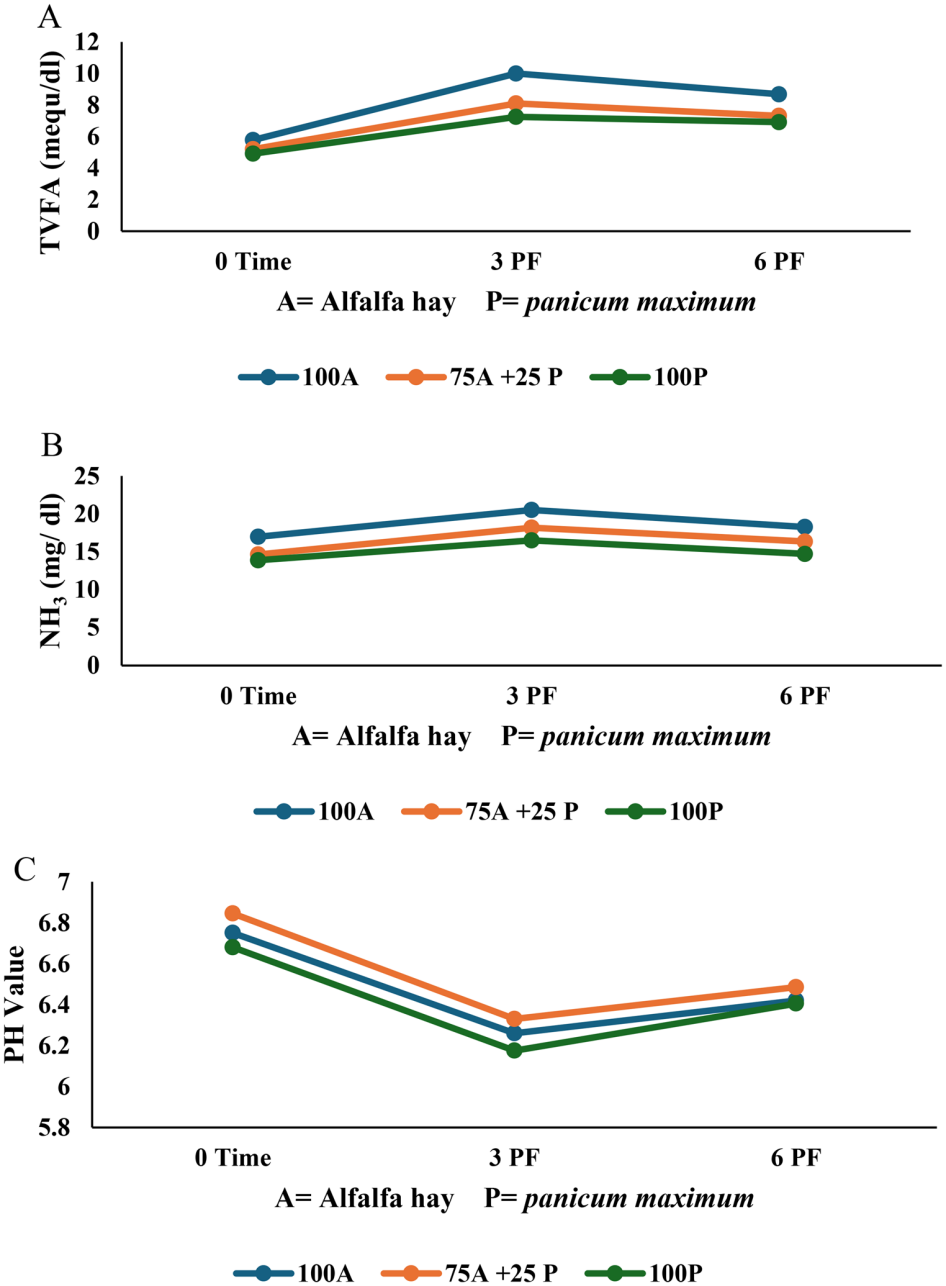
(**A**) The effect of substituting alfalfa hay with *Panicum maximum* hay on TVFA. **B**. The effect of substituting alfalfa hay with *Panicum maximum* hay on NH_3_. **C.** The effect of substituting alfalfa hay with *Panicum maximum* hay on PH

**Fig. 2:**
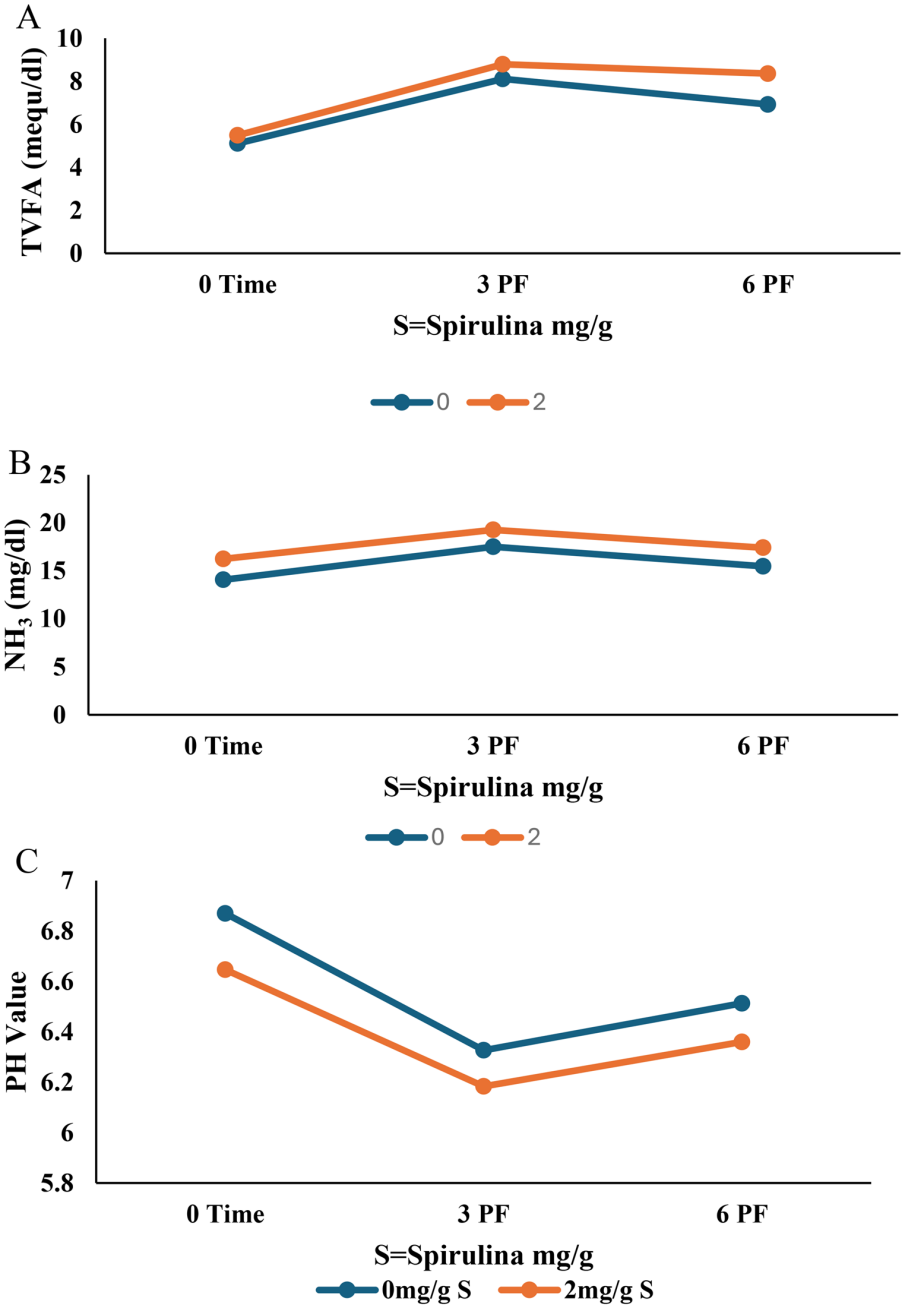
(**A**) The effect of spirulina addition on TVFA. (**B**) The effect of spirulina addition on NH_3_. (**C**) The effect of spirulina addition on PH

As shown in Table 3; Fig. 1A and B, diets containing 40% alfalfa hay exhibited significantly higher (*P* < 0.0001) TVFA and NH₃ concentrations compared with diets containing 30% alfalfa hay + 10% *Panicum maximum* hay or 40% *Panicum maximum* hay. Furthermore, Table 3; Fig. 2 A and 2B demonstrate that Spirulina supplementation at 2 mg/g significantly increased (*P* < 0.0001) TVFA and NH₃ concentrations compared to non-supplemented diets. Sampling time had a significant effect (*P* < 0.05), with the lowest TVFA and NH₃ values observed at 0 h (pre-feeding), reaching a plateau at 3 h post-feeding, and beginning to decline after 6 h from feeding. Notably, the combination of 40% alfalfa hay with 2 mg/g Spirulina (T2) yielded significantly higher (*P* < 0.0001) TVFA and NH₃ concentrations than diets containing 30% alfalfa + 10% *Panicum maximum* hay or 40% *Panicum maximum* hay, even when supplemented with Spirulina. The interaction between forage type, Spirulina supplementation, and sampling time was significant for both TVFA (*P* = 0.0165) and NH₃ (*P* = 0.0010). The highest values were observed in T2 at 3 h post-feeding, followed by T1 at the same time point, and T2 at 6 h. The lowest values were recorded in T5 at 0 h.

Forage substitution, Spirulina supplementation, and sampling time also showed a significant (*P* < 0.0001) but non-linear effect on ruminal pH (Table 3; Fig. 1C). The highest pH was observed at 0 h, followed by a decrease at 3 h post-feeding, and a subsequent increase at 6 h. The highest pH was recorded in T3 (30% alfalfa + 10% *Panicum maximum* hay without Spirulina), followed by T1, T5, T4, T2, and T6, respectively. Mixing alfalfa with *Panicum maximum* (T3) increased pH compared to diets containing 40% alfalfa (T1) or 40% *Panicum maximum* (T5). Spirulina supplementation led to a reduction in pH values, particularly in T4, followed by T2 and T6 (Table 3; Fig. 2C). The interaction between forage type, Spirulina supplementation, and sampling time was not significant (*P* > 0.05) for ruminal PH.

### Digestibility

**Fig. 3:**
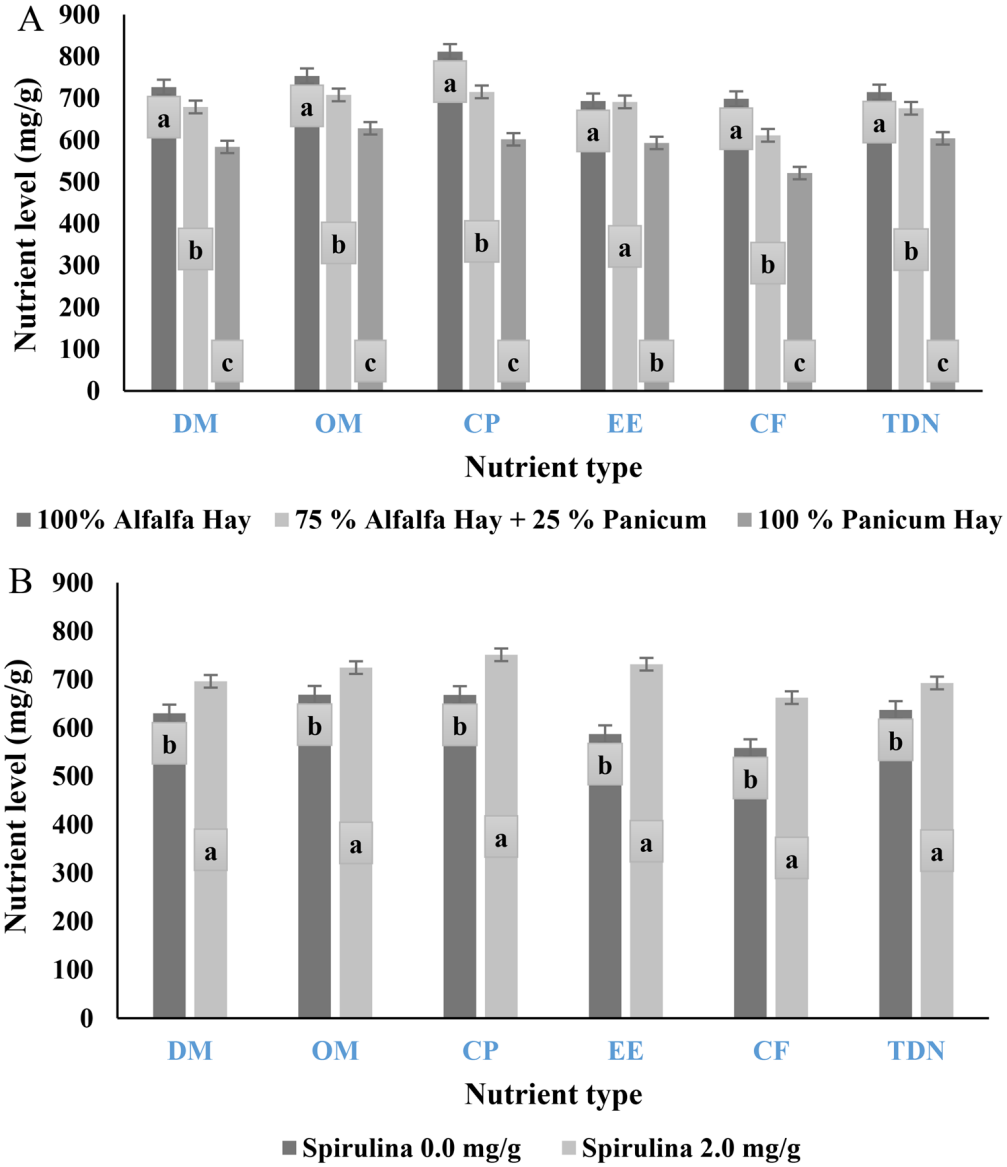
**A**: The effect of substituting alfalfa hay with *Panicum maximum* hay on nutrient composition of the diet. **B**: The effect of spirulina supplementation on the nutrient composition of the diet

The data in Table 4; Fig. 3A indicate that diets containing 40% alfalfa hay as roughage showed significantly (*P* < 0.0001) the highest nutrient digestibility and TDN compared to those with 10% panicum and 30% alfalfa hay. Conversely, the lowest values (*P* < 0.0001) were recorded for diets containing 40% *Panicum maximum*. The data also showed that animals receiving diets supplemented with 2.0 mg/g Spirulina had significantly higher (*P* < 0.0001) digestibility and improved nutritive value compared to animals that did not receive Spirulina supplementation (Fig. 3B). The interaction between diet and Spirulina supplementation had no significant effect (*P* > 0.05) on all digestibility and TDN, except for CP. Digestibility of crude protein (CP) was significantly higher for T2 (100% alfalfa hay with 2 mg/g Spirulina) and T1 (100% alfalfa hay), followed by T4 (75% alfalfa hay + 25% *Panicum maximum* with 2 mg/g Spirulina) and T3 (75% alfalfa hay + 25% *Panicum maximum*). Conversely, the lowest values were recorded for T5 (40% *Panicum maximum*) and T6 (100% *Panicum maximum* with 2 mg/g Spirulina).

**Table 4:** The effect of substituting alfalfa hay with *Panicum maximum* hay with or without spirulina addition on metabolic and nutritive value during metabolic trial

Forage contents	TRT	Spirulina, mg/g	DM, mg/g	OM, mg/g	CP, mg/g	EE, mg/g	CF, mg/g	TDN, mg/g
100% A	T1	0.0 mg/g	689.59	723.86	771.30^b^	624.33	637.58	685.69
	T2	2.0 mg/g	763.17	782.81	851.75^a^	762.39	759.53	743.43
75% A + 25% P	T3	0.0 mg/g	655.44	689.74	685.20^d^	621.61	562.97	657.25
	T4	2.0 mg/g	702.92	726.24	745.33^c^	760.88	659.70	694.70
100% P	T5	0.0 mg/g	544.81	591.88	547.60^f^	515.13	474.07	568.24
	T6	2.0 mg/g	622.37	664.46	655.80^e^	671.10	568.06	639.88
Standard Error			9.1713	8.3683	7.7307	12.2290	13.5845	7.9743
P value		Substituting	< 0.0001	< 0.0001	< 0.0001	< 0.0001	< 0.0001	< 0.0001
		Spirulina	< 0.0001	< 0.0001	< 0.0001	< 0.0001	< 0.0001	< 0.0001
		Interaction	0.2446	0.1356	0.0282	0.7218	0.5426	0.1402

A: Alfalfa Hay - P: Panicum Hay - S: Spirulina. T1 (Control): 0% *Panicum maximum* (40% alfalfa hay) + no Supplementation, T2: 0% *Panicum maximum* (40% alfalfa hay) + 2 mg/g Spirulina, T3: 25% *Panicum maximum* (30% alfalfa hay + 10% *Panicum maximum* hay) + no Supplementation, T4: 25% *Panicum maximum* (30% alfalfa hay + 10% *Panicum maximum* hay) + 2 mg/g Spirulina, T5: 100% *Panicum maximum* (40% *Panicum maximum* hay) + no Supplementation, T6: 100% *Panicum maximum* (40% *Panicum maximum* hay) + 2 mg/g Spirulina. TRT: Treatments. DM: Dry matter, OM: Organic matter, CP: Crude protein, EE: Ether extract, CF: Crude fiber, TDN: Total Digestible Nutrients

### Nitrogen balance

**Fig. 4:**
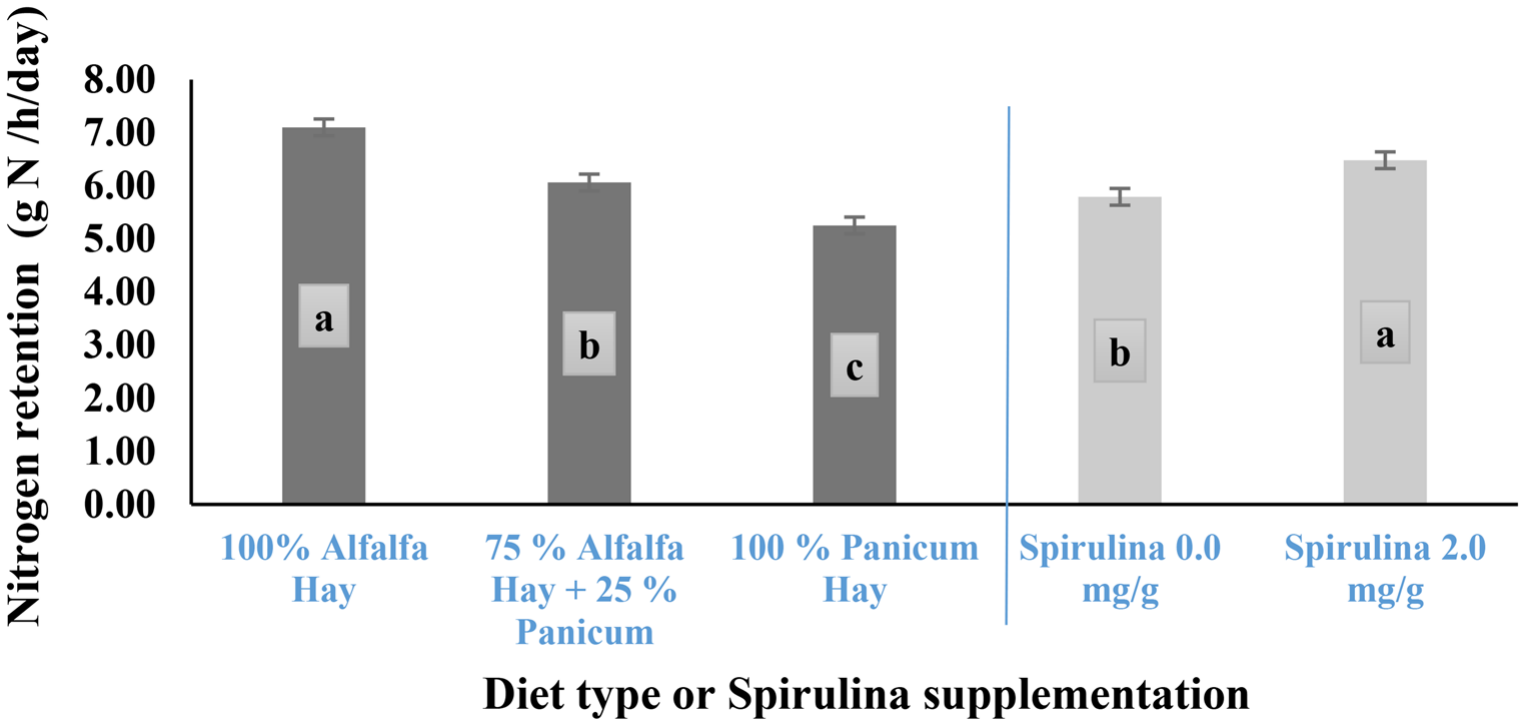
The effect of substituting alfalfa hay with *Panicum maximum* hay and spirulina on nitrogen balance

The data in Table 5; Fig. 4 showed that the effect of substituting alfalfa hay with *Panicum maximum* hay (at 0%, 25%, 100%) on nitrogen intake, urinary nitrogen, and total nitrogen were not significant (*P* > 0.05), However, the highest significant (*P* < 0.0001) values of fecal nitrogen were recorded for rams fed on diets containing *Panicum maximum* (40 and 10% with no significant differences between them), while the lowest fecal nitrogen was recorded for rams fed diets containing 40% alfalfa hay. As for the nitrogen balance, the highest values were recorded with rams fed 40% alfalfa, while the lowest values were recorded for rams fed on *Panicum maximum* (10 and 40% without significant differences).

**Table 5:** The effect of substituting alfalfa hay with panicum hay with or without spirulina addition on nitrogen balance (g/h/day) during metabolic trial

Forage contents	TRT	Spirulina, mg/g	TNI	FN	UN	TNE	NB
100% A	T1	0.0 mg/g	36.48	10.60	19.18	29.78	6.69
	T2	2.0 mg/g	39.70	9.82	22.37	32.19	7.51
75% A + 25% P	T3	0.0 mg/g	36.66	13.26	17.69	30.96	5.70
	T4	2.0 mg/g	39.82	11.67	21.72	33.39	6.43
100% P	T5	0.0 mg/g	36.49	11.37	20.11	31.49	5.00
	T6	2.0 mg/g	40.41	12.44	22.45	34.90	5.51
Standard Error		Interaction	1.4914	0.5917	1.0830	1.4979	0.2241
P value		Substituting	0.9719	0.0065	0.361	0.3687	< 0.0001
		Spirulina	0.0154	0.3862	0.0036	0.0441	0.0028
		Interaction	0.9611	0.1108	0.7439	0.9302	0.778

A: Alfalfa Hay - P: Panicum Hay - S: Spirulina. T1 (Control): 0% *Panicum maximum* (40% alfalfa hay) + no Supplementation, T2: 0% *Panicum maximum* (40% alfalfa hay) + 2 mg/g Spirulina, T3: 25% *Panicum maximum* (30% alfalfa hay + 10% *Panicum maximum* hay) + no Supplementation, T4: 25% *Panicum maximum* (30% alfalfa hay + 10% *Panicum maximum* hay) + 2 mg/g Spirulina, T5: 100% *Panicum maximum* (40% *Panicum maximum* hay) + no Supplementation, T6: 100% *Panicum maximum* (40% *Panicum maximum* hay) + 2 mg/g Spirulina. Nitrogen intake (TNI), Fecal nitrogen (FN), Urinary nitrogen (UN), Total nitrogen excretion (TNE), Nitrogen balance (NB). TRT: Treatments

Concerning the effect of Spirulina supplementation on nitrogen balance parameters, Fig. 4; Table 5 showed that nitrogen intake, urinary nitrogen, total nitrogen excretion, and nitrogen balance were significantly higher (*P* < 0.05) for rams that received rations supplemented with Spirulina compared to those that consumed rations free from any supplement. Conversely, no significant difference was recorded for fecal nitrogen excretion. The interaction between diet and spirulina supplementation was not significant (*P* > 0.05) on all parameters of nitrogen balance.

### Serum blood parameters

The data in Table 6 showed that the highest values (*P* < 0.0001) of total protein (TP), albumin (Alb), globulin (Gol), and urea were recorded in rams that received diets containing 40% alfalfa hay, followed by those that received diets of 10% panicum and 30% alfalfa hay. However, the highest values of creatinine (Creat), cholesterol (Chol), Triglycerides (TG), and ALT and AST activity were recorded for rams that were fed 40% *Panicum maximum* compared to rams fed 10% *Panicum maximum* and 30% alfalfa hay. In addition, the data indicate that rams receiving diets supplemented with 2 mg/g spirulina had higher TP, Alb, Glo, and urea compared to rams that did not receive the supplement, while Creatinine, cholesterol, Triglycerides, and ALT and AST activity were decreased with Spirulina addition (Table 7). Spirulina supplementation significantly increased all serum blood parameters (*P* < 0.05). The TP, Alb, Gol, and urea were highest in rams fed 40% alfalfa hay with 2 mg/g spirulina, compared with those fed 10% *Panicum maximum* + 30% alfalfa hay with 2 mg/g Spirulina. Serum data displayed a significant (*P* < 0.05) interaction between diet and Spirulina supplementation (Table 8), except for Alb and TG. Blood parameters of TP, Alb, Gol, and urea were negatively affected by substituting alfalfa with *Panicum maximum*, especially in the rams fed on 40% *Panicum maximum*. Spirulina supplementation at 2 mg/g improved the measured blood biochemical parameters across dietary treatments. The greatest improvement was observed in rams fed diets containing 10% *Panicum maximum* (Diet 2), whereas animals fed 40% *Panicum maximum* showed comparatively lower responses, regardless of Spirulina supplementation. However, the values recorded in rams fed 40% alfalfa hay (diet 1), with or without Spirulina, remained superior to those observed in all *Panicum maximum*–based diets.

**Table 6:** The effect of substituting alfalfa hay with *Panicum maximum* hay on blood parameters during metabolic trial

Forage contents	TP, g/dl	Alb, g/dl	Glo, g/dl	urea, mg/dl	Creat, mg/dl	Chol, mg/dl	TG, mg/dl	ALT, U/L	AST, U/L
100% A	7.30^a^	2.85^a^	4.44^a^	61.08^a^	0.88^c^	56.23^c^	31.19^c^	18.71^b^	91.65^b^
75% A + 25% P	7.14^b^	2.80^a^	4.33^b^	49.82^b^	0.95^b^	64.97^b^	33.52^b^	16.89^c^	83.13^c^
100% P	6.83^c^	2.71^b^	4.13^c^	46.31^c^	1.06^a^	71.61^a^	36.04^a^	20.01^a^	99.95^a^
Standard error	0.0283	0.0188	0.0258	0.3929	0.0133	0.3440	0.7685	0.1724	0.8542
P value	< 0.0001	0.0004	< 0.0001	< 0.0001	< 0.0001	< 0.0001	< 0.0001	< 0.0001	< 0.0001

A: Alfalfa Hay - P: Panicum Hay - S: Spirulina. T1 (Control): 0% *Panicum maximum* (40% alfalfa hay) + no Supplementation, T2: 0% *Panicum maximum* (40% alfalfa hay) + 2 mg/g Spirulina, T3: 25% *Panicum maximum* (30% alfalfa hay + 10% *Panicum maximum* hay) + no Supplementation, T4: 25% *Panicum maximum* (30% alfalfa hay + 10% *Panicum maximum* hay) + 2 mg/g Spirulina, T5: 100% *Panicum maximum* (40% *Panicum maximum* hay) + no Supplementation, T6: 100% *Panicum maximum* (40% *Panicum maximum* hay) + 2 mg/g Spirulina. Total protein (TP), Albumin (Alb), Globulin (Glo), Urea (urea), Creatinine (Creat), Cholesterol (Chol), Triglycerides (TG), Alanine aminotransferase (ALT), Aspartate aminotransferase (AST). TRT: Treatments

**Table 7:** The effect of spirulina supplementation on blood parameters during metabolic trial

Spirulina, mg/g	TP, g/dl	Alb, g/dl	Glo, g/dl	urea, mg/dl	Creat, mg/dl	Chol, mg/dl	TG, mg/dl	ALT, U/L	AST, U/L
0.0 mg/g	6.82^b^	2.74^b^	4.07^b^	50.50^b^	1.00^a^	67.22^a^	36.18^a^	19.66^a^	99.60^a^
2.0 mg/g	7.36^a^	2.84^a^	4.52^a^	54.30^a^	0.93^b^	61.33^b^	30.98^b^	17.41^b^	83.55^b^
Standard error	0.0231	0.0154	0.0211	0.3208	0.0109	0.2809	0.6275	0.1408	0.6974
P value	< 0.0001	0.0008	< 0.0001	< 0.0001	0.0005	< 0.0001	< 0.0001	< 0.0001	< 0.0001

A: Alfalfa Hay - P: Panicum Hay - S: Spirulina. T1 (Control): 0% *Panicum maximum* (40% alfalfa hay) + no Supplementation, T2: 0% *Panicum maximum* (40% alfalfa hay) + 2 mg/g Spirulina, T3: 25% *Panicum maximum* (30% alfalfa hay + 10% *Panicum maximum* hay) + no Supplementation, T4: 25% *Panicum maximum* (30% alfalfa hay + 10% *Panicum maximum* hay) + 2 mg/g Spirulina, T5: 100% *Panicum maximum* (40% *Panicum maximum* hay) + no Supplementation, T6: 100% *Panicum maximum* (40% *Panicum maximum* hay) + 2 mg/g Spirulina. Total protein (TP), Albumin (Alb), Globulin (Glo), Urea (urea), Creatinine (Creat), Cholesterol (Chol), Triglycerides (TG), Alanine aminotransferase (ALT), Aspartate aminotransferase (AST). TRT: Treatments

**Table 8:** The effect of substituting alfalfa hay with *Panicum maximum* hay with or without spirulina addition on blood parameters during metabolic trial

Forage contents	TRT	Spirulina, mg/g	TP, g/dl	Alb, g/dl	Glo, g/dl	urea, mg/dl	Creat, mg/dl	Chol, mg/dl	TG, mg/dl	ALT, U/L	AST, U/L
100% A	T1	0.0 mg/g	6.97^c^	2.81	4.16^d^	57.18^b^	0.90^cd^	58.07^d^	33.84	20.24^b^	94.31^c^
	T2	2.0 mg/g	7.64^a^	2.91	4.73^a^	64.98^a^	0.87^d^	54.40^e^	28.55	17.20^d^	89.00^d^
75% A + 25% P	T3	0.0 mg/g	6.85^d^	2.75	4.10^d^	48.96^d^	0.98^b^	66.98^b^	36.51	17.39^d^	98.13^b^
	T4	2.0 mg/g	7.43^b^	2.87	4.56^b^	50.69^c^	0.94^cb^	62.98^c^	30.54	16.39^e^	68.13^e^
100% P	T5	0.0 mg/g	6.64^e^	2.68	3.97^e^	45.38^e^	1.14^a^	76.62^a^	38.21	21.37^a^	106.37^a^
	T6	2.0 mg/g	7.03^c^	2.75	4.29^c^	47.25^d^	0.99^b^	66.62^b^	33.88	18.66^c^	93.54^c^
Standard error			0.0400	0.0266	0.0365	0.5557	0.0188	1.0868	0.4865	0.2438	1.2080
P value			0.0132	0.6171	0.0164	0.0002	0.0127	0.0215	0.2761	0.0028	< 0.0001

A: Alfalfa Hay - P: Panicum Hay - S: Spirulina. T1 (Control): 0% *Panicum maximum* (40% alfalfa hay) + no Supplementation, T2: 0% *Panicum maximum* (40% alfalfa hay) + 2 mg/g Spirulina, T3: 25% *Panicum maximum* (30% alfalfa hay + 10% *Panicum maximum* hay) + no Supplementation, T4: 25% *Panicum maximum* (30% alfalfa hay + 10% *Panicum maximum* hay) + 2 mg/g Spirulina, T5: 100% *Panicum maximum* (40% *Panicum maximum* hay) + no Supplementation, T6: 100% *Panicum maximum* (40% *Panicum maximum* hay) + 2 mg/g Spirulina. Total protein (TP), Albumin (Alb), Globulin (Glo), Urea (urea), Creatinine (Creat), Cholesterol (Chol), Triglycerides (TG), Alanine aminotransferase (ALT), Aspartate aminotransferase (AST). TRT: Treatments

The highest concentrations of creatinine, cholesterol, triglycerides, and liver enzymes (ALT and AST) were recorded in rams fed diets containing 40% *Panicum maximum*. Supplementation with Spirulina at 2 mg/g resulted in a slight reduction in these parameters. In contrast, the inclusion of Spirulina in diets containing 40% alfalfa hay or 10% *Panicum maximum* was associated with lower values of these biochemical indices.

## Discussion

### Rumen fermentation parameters

Rumen NH₃ and TVFA concentrations exhibited a typical pattern, being lowest at 0 h (pre-feeding) and peaking at 3 h post-feeding. This pattern is likely due to the fermentation process of both nonstructural and structural carbohydrates, as well as the degradable protein sources, which start at a low rate when the substrate is absent, increase over time, reach the maximum value at 3 h after feeding, and then decrease until the next meal. This pattern aligns with the gradual disappearance of the substrate as described by Venkateswarlu et al. (2013).

The higher NH_3_ and TVFA in rumen liquor of animals that received diets with 40% alfalfa hay, compared to those that received diets with 10% Panicum and 30% alfalfa hay, as well as those that received 40% *Panicum maximum*, may be attributed to lower levels of ADF and NDF, and Ash contents in alfalfa hay as compared to *Panicum maximum*. According to Miller et al. (2021), forage fiber affects ruminal fermentation, nutrient digestibility, and turnover due to its physical and chemical characteristics. Additionally, Wang et al. (2020) report that carbohydrates can be fermented by a variety of bacteria in the rumen and converted into TVFA by corresponding enzymes.

Rams receiving rations supplemented with 2 mg/g Spirulina exhibited significantly higher TVFA and NH_3_ concentrations in rumen fluid compared to unsupplemented groups. Spirulina is nutrient-dense and can stimulate extracellular enzyme secretion by gut microflora (Tovar et al. 2002), enhancing the digestion of fiber, carbohydrates, and proteins, which subsequently increases the concentration of TVFA and ammonia. Additionally, Gaafar et al. (2017) observed that adding Spirulina to cows’ drinking water increased TVFA concentrations in a dose-dependent manner. The increase in NH_3_ with substitution and Spirulina inclusion may be due to the higher crude protein content in alfalfa compared to *Panicum maximum*, and to the enhanced crude protein digestibility as observed in T2 (100% alfalfa hay with Spirulina supplementation). Ammonia production in ruminants is influenced by both the concentration and utilization efficiency of rumen-degradable protein (Kaasik et al. 2009). This was confirmed by Xia et al. (2018), who found that dietary crude protein is crucial for promoting ruminal fermentation and nutrient digestibility and leads to an increment in the concentration of ammonia in the rumen. Also, adding Spirulina to the diet increases the ammonia concentration significantly. This may be attributed to the effect of Spirulina as algae containing a rich source of functional metabolites such as proteins, peptides, amino acids, polysaccharides, lipids, minerals, and polyphenols (Brown et al. 2014). Panjaitan et al. (2014) also found increased ammonia concentration in cannulated Bos indicus steers with increased Spirulina (0, 0.5, 1.4, 2.5, and 6.1 g DM/kg) inclusion in the diet.

Rumen pH followed the typical post-prandial diurnal pattern: it was highest before feeding, decreased at 3 h post-feeding, and then increased again at 6 h. The observed changes are consistent with Zeid et al. (2011), who reported that pH values were initially high before feeding due to minimal fermentation activity, decreased after feeding due to soluble carbohydrates being rapidly fermented and TVFA accumulated, and then increased again after 6 h as TVFA were absorbed across the rumen wall and saliva bicarbonate helped buffer the rumen environment. Additionally, the gradual increase in pH between 3 and 6 h post-feeding is associated with reduced availability of fermentable substrates, enhanced TVFA absorption, and increased salivary secretion during rumination, all of which contribute to rumen buffering (Beauchemin et al., 2003; Plaizier et al. 2008).

Rumen pH was lower in animals fed Spirulina-supplemented diets, likely due to increased TVFA and NH₃ production. These results were consistent with Osman et al. (2015), who reported that pH values were affected by varied factors such as drinking water, ruminal TVFA, and NH_3_. Spirulina supplementation resulted in lower pH values. This is in harmony with Riad et al. (2019), who found that the pH value was significantly lower in rumen liquor with Spirulina additive compared with that of the control. Christodoulou et al. (2023) found a decrease in rumen pH values with increasing the concentration of Spirulina in the drinking water of cattle, while Wang et al. (2023) reported no changes in the ruminal pH value of lambs supplemented with Spirulina.

### Digestibility

Although the diets were formulated to be isonitrogenous and isocaloric, a significant decrease (*P* < 0.0001) in the digestibility of DM, OM, CP, EE, and CF was observed because of substituting alfalfa with *Panicum maximum* hay. This can be attributed to the increase in crude fiber and ash content resulted from the increasing replacement rate of *Panicum maximum* hay. These results were consistent with Lu et al. (2019), who reported that the inclusion of dietary protein in supplements enhances the availability of protein to rumen bacteria, allowing for accelerated digestion. Cilliers and Van der Merwe (1993) explained that a decrease in *Panicum maximum* digestibility was associated with a reduction in N content and an increase in ADF, NDF, and ADL contents. Also, higher contents of total phenols and tannins in *Panicum maximum* compared to alfalfa can form a complex of proteins and tannins that prevents the protein from reaching ruminal microbes, and sometimes this complex prevents the post-ruminal digestion and absorption of proteins (Moheghi et al., 2022). A similar trend was observed by Kumar (1992), who found that secondary metabolites or anti-nutritional factors (ANFs) in animal diets negatively affected nutrient digestion and absorption, as the studied *Panicum maximum* plant contained high tannins. The increase in nutrient digestibility with Spirulina supplementation has been attributed to the fact that Spirulina was considered a source of protein, vitamins, minerals, carotene, xanthophyll, and many other biologically important compounds that positively affect nutrient digestibility (Kulpys et al., 2009; Lafarga et al. 2020; Chia et al. 2019). Similar results were recorded by Hassanien et al. (2015); they recorded significant improvements in DM and OM digestibility coefficients as well as the nutritional value of the diets when supplementing lactating Damascus goats with two types of algae (Spirulina and Ulva rigida) at 0.2% of the feed intake. Moreover, the addition of Spirulina led to the activation of microflora in the rumen by providing it with mineral elements, vitamins, and fatty acids, especially Omega-3. As a result, fiber digestion and, consequently, TDN values increase. The values of TDN were different among animal groups because of the different chemical compositions and nutrient digestibility of diets.

### Nitrogen balance

The observed decrease in nitrogen balance with increasing levels of *Panicum maximum* hay is mainly due to its lower crude protein digestibility compared to alfalfa, which consequently reduces nitrogen retention. As reported by Mupangwa et al. (2000), forage with lower CP and higher structural fiber constituents (NDF and ADF) limits ruminal nutrient digestibility and decreases energy availability for microbial protein synthesis. *Panicum maximum* typically contains more fiber and less degradable protein than alfalfa, resulting in reduced rumen-degradable nitrogen and lower microbial growth efficiency (Van Soest 1994; Pathak 2008). Additionally, the higher concentration of tannins and phenolic compounds in *Panicum maximum*, compared to alfalfa hay, contributes to the formation of tannin–protein complexes, which decrease rumen degradability and post-ruminal digestibility of dietary protein (Moheghi et al. 2022; Min et al. 2003). This mechanism helps explain the superior nitrogen retention observed in animals fed alfalfa-based diets, as higher CP intake and improved ruminal fermentation are positively associated with enhanced nitrogen utilization (Osman et al. 2015). Consistent with these findings, Hassan et al. (2022) reported that Barki rams fed diets containing 100% *Panicum maximum* had the lowest nitrogen intake and nitrogen balance values, confirming the negative impact of high-Panicum diets on nitrogen utilization. The positive effect of Spirulina on the nitrogen balance was due to several factors, including the high nutrient density of Spirulina and the stimulation of extracellular enzyme secretion by gut microflora (Tovar et al. 2002). Spirulina also contains carbohydrates, which help increase microbial protein synthesis (Panjaitan et al. 2014).

### Serum blood parameters

All the studied blood parameters showed differences between treatments, but all were within the normal range for healthy sheep according to Otesile et al. (1991). The increase in total protein in the blood serum of animals fed on alfalfa hay, especially those with added algae, may be due to the increase in protein in these diets compared to other treatments fed on *panicum maximum* hay, as total protein in the blood serum reflects the crude protein in the diets, and this is consistent with Yousuf et al. (2007). *Panicum maximum* addition decreased total protein, albumin, globulin, and urea. This may be attributed to the tannin contents of *Panicum maximum;* similar results were observed by Vasta et al. (2009), who showed a reduction in serum total protein concentration by adding tannins to the diet. The increase in serum urea in animals fed on alfalfa, especially those fed with added algae, may be due to the increase in crude protein in diets, as urea is produced by the breakdown of proteins and is excreted in the urine. Urea is often used to evaluate renal function in animals. Urea nitrogen level is affected by liver function, diet, intestinal absorption, and hydration (Melillo 2007). The concentration of urea is artificially increased by a high-protein diet (Liu et al. 2016).

In the present study, the elevation in creatinine, cholesterol, triglycerides, ALT, and AST values in the rams fed *Panicum maximum* hay, may be due to decreased fiber digestibility, where, fiber digestion in the digestive tract involves binding of most of the bile salts for excretion, once most bile salts are depleted, the body must synthesize new bile salts derived from the body’s cholesterol (Hatta et al. 2019). The more fiber digested, the more efficiently the fiber binds the bile salts, which may lead to reduced cholesterol in the body. (Hatta et al. 2019). In addition, certain dietary fibers have been reported to lower cholesterol by binding bile acids and reducing their recycling through the enterohepatic circulation. In addition, certain fibers may delay the digestion and absorption of fat (Ebihara and Schneeman 1989), unlike what happened with *Panicum maximum* hay. Since almost half of the cholesterol needs come from the biosynthesis process that takes place in the intestines, skin, and especially in the liver (about 50%) (Hatta et al. 2019), the cholesterol level increased with Panicum hay may explain the increase in liver enzymes (ALT and AST).

Regarding the effect of Spirulina on blood proteins and urea. Total protein, albumin, globulin, and urea increased with Spirulina supplementation may be due to the high crude protein in Spirulina (Assar et al. 2023). Furthermore, the findings of Hassanien et al. (2015) showed that supplemented rations with Spirulina tended to have higher total protein, albumin, and urea, with no significant differences. Additionally, Khalifa et al. (2016) supplemented dairy goat rations with Spirulina at a dosage of 0.5 g per head per day. This supplementation resulted in decreased levels of serum cholesterol, triglycerides, ALT, and AST.

These findings may be related to the increased saponin content of Spirulina, which can be explained by Matsuura (2001), who reported that saponin and bile acids can form large mixed micelles, which increase the excretion of bile acids, thus accelerating cholesterol metabolism in the liver, and then reducing serum cholesterol. Also, Spirulina has been reported to have the C phycocyanin protein, which inhibits pancreatic lipase activity (Torres-Duran et al. 2007), and it has antioxidant properties, hypolipidemic action, and anti-inflammatory effects (Bashandy et al. 2011).

Overall, in line with the study’s objective, the results clearly demonstrate that replacing 25% of alfalfa hay with *Panicum maximum* combined with Spirulina supplementation improved rumen fermentation parameters and nutrient utilization compared to diets containing 100% *Panicum maximum*. However, these improvements did not exceed those observed with diets containing 100% alfalfa hay. This indicates that partial substitution, when combined with Spirulina, can be a practical strategy under forage-limited conditions, supporting the hypothesis that such combinations enhance digestibility and metabolic responses in Barki rams.

## Conclusion

Partial substitution of alfalfa hay with *Panicum maximum* hay at a 25% replacement rate improved roughage quality and digestibility compared with using *Panicum maximum* as the sole source of roughage. Incorporating Spirulina as a feed additive (2 mg/g) provides an effective strategy to improve the quality of *Panicum maximum* and alfalfa hay, which can be used to maximize the benefits of experimental diets.

## Data Availability

The datasets generated and analyzed during the current study will be provided upon reasonable request from the corresponding author.
